# Formulation and Release Kinetics of Neomycin Sulfate from a Polymeric Oil-in-Water Emulgel for Topical Application: A Proof-of-Concept Study

**DOI:** 10.3390/gels12090758

**Published:** 2026-08-24

**Authors:** Alexandra Virginia Bounegru, Iulian Bounegru, Simona Butan

**Affiliations:** 1 Department of Chemistry, Physics and Environment, Faculty of Sciences and Environment, “Dunărea de Jos” University, 47 Domneasca Street, 800008 Galati, Romania; simona.patriche@ugal.ro; 2 REXDAN Research Infrastructure, “Dunărea de Jos” University, 98 George Coșbuc Street, 800385 Galati, Romania; 3 Competences Centre—Interfaces-Tribocorrosion-Electrochemical Systems (CC-ITES), “Dunărea de Jos” University of Galati, 47 Domneasca Street, 800008 Galati, Romania; iulian.bounegru@ugal.ro

**Keywords:** chitosan, antibiotic, diffusion, polymer matrix

## Abstract

This proof-of-concept study aimed to develop and characterize a polymeric oil-in-water emulgel containing neomycin sulfate for topical application, using chitosan and methylcellulose as gelling agents, Tween 80 as emulsifier, and sweet almond oil as the oily phase. The novelty of the study lies in the formulation of a chitosan–methylcellulose oil-in-water emulgel for a highly hydrophilic aminoglycoside antibiotic, together with the integrated evaluation of its microstructure, rheological behavior, hydration capacity, and release kinetics. The formulation was evaluated in terms of physicochemical properties, microstructure, rheological behavior, short-term stability, drug content, and in vitro release performance. Rheological analysis showed pseudoplastic behavior, with apparent viscosity decreasing as the rotational speed increased, indicating suitable spreadability under applied stress. The neomycin sulfate content was 99.1 ± 0.7%, demonstrating efficient incorporation of the active substance. In vitro release testing showed a progressive release profile, reaching 70.1% of the theoretical drug content after 120 min. Kinetic analysis showed that the zero-order model gave the best empirical fit over the interval studied (R^2^ = 0.9980), while the Korsmeyer–Peppas exponent varied with the sampling points included and was therefore not used to support a specific transport mechanism. Because a single drug-loaded formulation was evaluated, without a placebo emulgel, a free-drug solution, or a comparator, the release profile cannot be attributed to the polymeric network as such; the results provide a preliminary basis for the further development of chitosan–methylcellulose emulgels as topical vehicles for neomycin sulfate.

## 1. Introduction

Topical drug delivery represents an important approach for the treatment of localized skin conditions, as it allows the active pharmaceutical ingredient to be applied directly at the affected site while reducing systemic exposure and the risk of systemic adverse effects. Among topical dosage forms, semisolid systems such as gels, creams, ointments, and emulgels are widely used because they can provide adequate residence time, ease of application, and patient acceptability [1]. Recent reviews have also emphasized the increasing interest in emulgels as topical drug delivery platforms due to their ability to combine the advantages of emulsions and gel-based matrices [2,3]. However, the therapeutic performance of these formulations depends not only on the concentration of the active substance but also on its distribution within the vehicle, its physicochemical properties, and the ability of the formulation matrix to control drug release.

Neomycin sulfate is an aminoglycoside antibiotic commonly used in topical pharmaceutical preparations due to its broad antibacterial activity against several Gram-negative and Gram-positive microorganisms [4,5]. Structurally, neomycin contains multiple amino and hydroxyl groups, which confer a strongly hydrophilic and polycationic character. The protonation of amino groups, with pKa values reported in the physiological range, favors high aqueous solubility and strong interactions with negatively charged biological targets [6,7]. Although these properties are essential for its antibacterial activity, they also influence its formulation behavior. Because of its highly polar and hydrophilic character, neomycin sulfate is expected to be preferentially associated with the aqueous domains of oil-in-water systems; therefore, its release from semisolid matrices may be strongly influenced by the structure and diffusional resistance of the polymeric network [8,9,10].

The development of suitable topical vehicles for neomycin sulfate therefore requires formulation strategies that maintain homogeneity, improve local retention, and modulate drug release. Emulgels are hybrid semisolid systems that combine the advantages of emulsions and gels. In oil-in-water emulgels, the oily phase is dispersed within an aqueous continuous phase, while the gel network increases viscosity and improves the physical stability of the dispersed system [11,12]. Compared with conventional emulsions, emulgels generally exhibit greater consistency, reduced phase separation, improved spreadability, and more favorable sensory characteristics for topical administration. Moreover, the presence of both aqueous and oily domains allows the incorporation of active substances with different solubility profiles, while the polymeric gel network can act as a diffusional barrier controlling drug transport [13,14]. Chitosan is a natural cationic polysaccharide obtained by deacetylation of chitin and has attracted considerable interest in food, nutraceutical, and especially pharmaceutical products due to its biocompatibility, biodegradability, film-forming ability, and bioadhesive properties [15,16,17,18]. Under acidic conditions, the amino groups of chitosan become protonated, allowing the polymer to form hydrated networks and interact with other formulation components [19]. In topical systems, chitosan can contribute to increased viscosity, improved retention at the application site, and partial control of active substance diffusion through the semisolid matrix [20,21]. The association of chitosan with methylcellulose, a hydrophilic cellulose derivative, may further improve the structure of the gel phase by increasing water retention and reducing molecular mobility within the formulation. Although several experimental studies have reported the formulation and evaluation of topical emulgels containing different active pharmaceutical ingredients, such as hesperetin, fluconazole, or gallic acid, these studies have generally focused on formulation optimization, physicochemical characterization, stability, and in vitro release performance [22,23,24]. However, limited attention has been given to oil-in-water emulgels designed for highly hydrophilic and ionizable aminoglycoside antibiotics, whose release is expected to be strongly influenced by their preferential localization in the aqueous phase and by the diffusional resistance of the polymeric network.

Therefore, the present study reports the development of a chitosan–methylcellulose oil-in-water emulgel designed for neomycin sulfate, together with an integrated evaluation of its physicochemical properties, microstructure, rheological behavior, hydration capacity, short-term stability, drug content, and in vitro release profile. The work is presented as a proof-of-concept investigation of a single prototype formulation. Since no placebo emulgel, free-drug solution, or comparator formulation was prepared, the design does not allow the contribution of the polymeric network to be separated from that of the remaining formulation components or of the diffusion membrane; the release behavior is therefore described rather than mechanistically assigned.

## 2. Results and Discussion

### 2.1. Organoleptic and Macroscopic Evaluation of the Emulgel

The prepared neomycin sulfate-loaded emulgel exhibited a homogeneous, semisolid, creamy appearance, with a white color characteristic of oil-in-water semisolid systems. No visible phase separation, large aggregates, or non-uniform areas were observed after preparation, indicating an adequate dispersion of the oily phase within the hydrophilic polymeric matrix. The formulation exhibited a faint characteristic odor, most likely attributable to the presence of sweet almond oil and acetic acid used for chitosan solubilization. The consistency was soft, gelatinous, and moderately viscous, allowing easy sampling and uniform spreading (Table 1). Upon application, the formulation formed a continuous layer, suggesting suitable spreadability and acceptable macroscopic properties for topical administration.

The observed semisolid structure can be attributed to the combined contributions of chitosan and methylcellulose, which form a hydrated polymeric network, while the oily phase may improve the formulation's sensory characteristics and emollient properties. Overall, the macroscopic evaluation indicated that the developed emulgel had appropriate organoleptic characteristics for topical use, with good homogeneity and no visible signs of physical instability immediately after preparation.

### 2.2. pH and Electrical Conductivity

The pH of the emulgel, determined after dispersion in distilled water at a 1:10 ratio, was 4.44 ± 0.03. This value therefore characterizes the diluted aqueous dispersion and not the undiluted emulgel; the same applies to the conductivity value reported below. This slightly acidic value is mainly attributable to acetic acid, which was used to solubilize chitosan by protonating its amino groups [25]. The pH is relevant for topical formulations, since the skin surface is naturally slightly acidic and topical products should remain within a range compatible with the cutaneous environment [26]. Therefore, the obtained pH suggests that the emulgel may be suitable for topical application. In addition, the acidic medium helps maintain chitosan in its protonated form, supporting its solubility and organization within the polymeric network.

The electrical conductivity of the emulgel dispersion was 267 µS/cm, indicating the presence of mobile ionic species in the aqueous phase. This conductivity may be related to the ionizable nature of neomycin sulfate, the presence of acetic acid, and the protonated amino groups of chitosan, confirming the ionic character of the emulgel matrix.

### 2.3. Optical Microscopy Analysis

**Figure 1:**
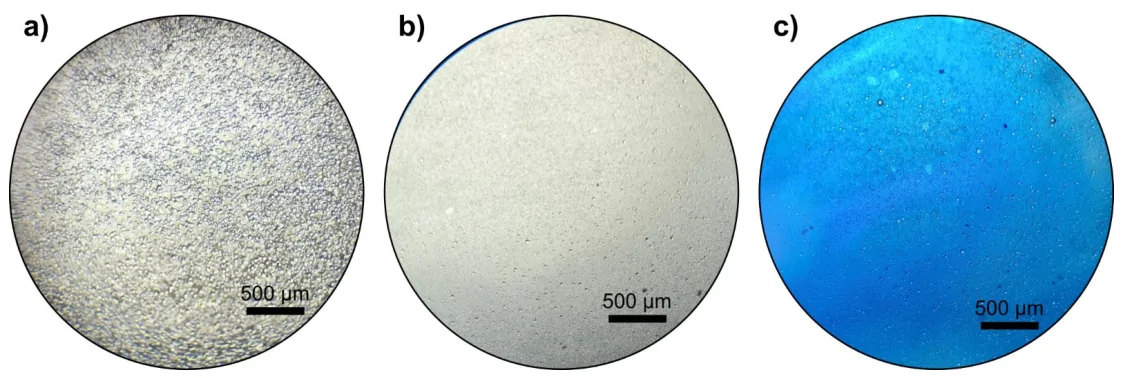
Optical micrographs of the neomycin sulfate-loaded emulgel (40×): (**a**) before ultrasonication, showing a heterogeneous distribution of dispersed droplets of variable size; (**b**) after ultrasonication, showing a finer and more uniform dispersion; (**c**) after staining with methylene blue, confirming the oil-in-water character of the emulgel through preferential staining of the continuous aqueous phase.

Optical microscopy was used to evaluate the microstructural organization of the neomycin sulfate-loaded emulgel and the effect of ultrasonication on the dispersion of internal phases. Before ultrasonication, the formulation exhibited a heterogeneous structure, with dispersed droplets/particles of variable size, indicating incomplete homogenization of the oily phase within the aqueous chitosan–methylcellulose matrix. After ultrasonication, the dispersed domains appeared smaller and more uniformly distributed, suggesting improved phase dispersion and structural homogeneity. Representative micrographs recorded before and after ultrasonication, together with the methylene blue staining test, are presented in Figure 1. As no quantitative droplet size analysis was performed, these descriptions are reported as qualitative observations. This effect can be attributed to the mechanical energy generated during ultrasonication, which promotes droplet disruption and distribution within the polymeric network [27].

Methylene blue staining confirmed the oil-in-water nature of the emulgel, as the hydrophilic dye was preferentially distributed in the aqueous continuous phase. Given the hydrophilic and ionizable nature of neomycin sulfate, the drug is expected to be primarily associated with the aqueous phase. Overall, the microscopic analysis shows that ultrasonication improved the uniformity of the dispersed phase, a feature relevant for physical stability, drug distribution, and reproducibility of the in vitro release profile.

### 2.4. Hydration Capacity of the Emulgel

The hydration capacity of the neomycin sulfate-loaded oil-in-water emulgel was evaluated to assess the interaction between the semisolid polymeric matrix and the aqueous receptor medium. The hydration index values of the emulgel at different contact times with the receptor medium are presented in Table 2.

Since the formulation contains an aqueous continuous phase structured by chitosan and methylcellulose, the observed gravimetric changes should be interpreted as additional water uptake rather than classical swelling of a dry hydrogel. The progressive increase in the hydration index indicates that the polymeric network can absorb and retain a moderate amount of the receptor medium, likely due to the hydrophilic groups present in both polymers. This additional hydration may increase polymer chain mobility, favoring matrix relaxation and partial structural reorganization during the release process. Similar behavior has been described for hydrophilic polymeric matrices, where water penetration and swelling influence drug dissolution, diffusion through the hydrated gel layer, and overall release kinetics [28,29]. Therefore, the hydration capacity results support the kinetic interpretation of neomycin sulfate release, suggesting that the process is governed not only by diffusion, but also by hydration-related relaxation of the chitosan–methylcellulose matrix.

### 2.5. Rheological Behavior

The rheological behavior of the emulgel was evaluated by measuring the apparent viscosity at different rotational speeds. The apparent viscosity decreased progressively as the rotational speed increased, from 1.032 Pa·s at 50 rpm to 0.572 Pa·s at 100 rpm (Table 3). This decrease indicates a pseudoplastic, shear-thinning behavior.

### 2.6. Spreadability

A 0.1 g emulgel sample had an initial diameter of 0.6 ± 0.1  cm and a contact area of 0.28 ± 0.09  cm^2^. After applying a 50 g load for 1 min, the diameter increased to 1.9 cm and the spreading area to 2.84 cm^2^, corresponding to an approximately 3.2-fold increase in diameter and a 10-fold increase in contact area. The estimated final film thickness was approximately 0.35 mm.

### 2.7. UV–Vis Calibration Curve for Neomycin Sulfate

A UV–Vis calibration curve was developed for the quantitative determination of neomycin sulfate released from the emulgel. Standard solutions were prepared in phosphate buffer, pH 7, over the concentration range of 5–100 µg/mL, and absorbance was measured at 300 nm. The relationship between absorbance and concentration was described by the equation A = 0.002827C+ 0.033222, R^2^ = 0.9905, *n* = 8. The calculated LOD and LOQ values were 0.128 µg/mL and 0.388 µg/mL, respectively. Both were obtained from the standard deviation of replicate blank readings and the slope of the calibration curve (Section 4.11), and both lie more than an order of magnitude below the lowest calibration standard, 5 µg/mL. They were therefore obtained by extrapolation and were not verified by measurement at or near those concentrations, as doing so would require preparing and repeatedly analyzing standards in that region. They are reported as calculated values only, are not presented as validated performance characteristics of the method, and are not used to support quantification below the calibration range.

**Figure 2:**
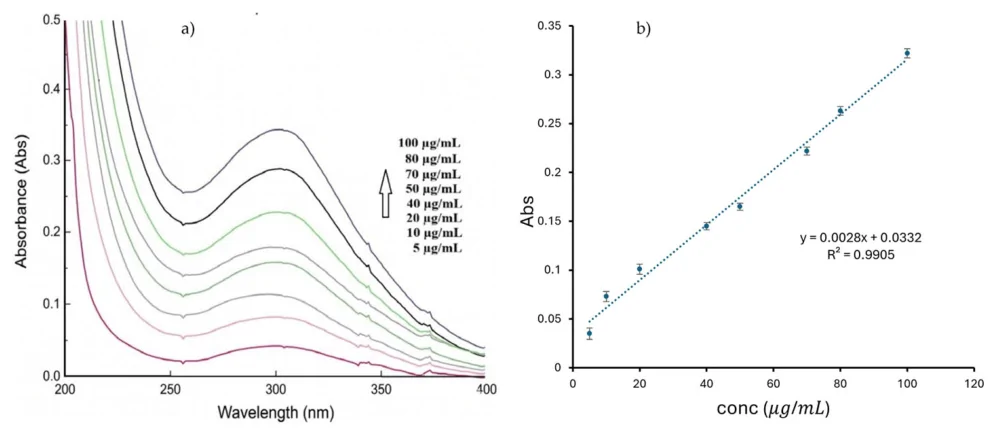
UV–Vis spectrophotometric analysis of neomycin sulfate in phosphate-buffered solution, pH 7.0: (**a**) absorption spectra of the standard solutions (5 to 100 µg/mL)showing a local absorption maximum at 300 ± 3 nm (mean ± SD, *n* = 8); (**b**) calibration curve constructed at 300 nm; data are expressed as mean ± SD (*n* = 3).

The spectra recorded between 200 and 400 nm showed a reproducible local maximum at 300 ± 3 nm, preceded by a minimum at approximately 256 nm (Figure 2a). This spectral shape supports attributing the signal to an analyte absorption band rather than to a simple light-scattering artifact. Phosphate buffer(pH 7.0)was used as the blank for both standards and samples. However, the non-zero intercept indicates a background contribution at 300 nm.

Possible spectral contributions from formulation excipients or components released from the inner eggshell membrane cannot be excluded. Tween 80, sweet almond oil, chitosan, and methylcellulose may contribute to absorbance or light scattering at 300 nm, while filtration may not completely remove submicron droplets or dissolved components. All drug-content, stability, and release samples were nevertheless prepared and measured under consistent analytical conditions and quantified using the same calibration curve. Therefore, the reported values are considered apparent concentrations suitable for internal comparison within the present study rather than validated absolute assay values.

### 2.8. Neomycin Sulfate Content

The apparent neomycin sulfate content of the emulgel, determined against the calibration curve, was 99.1 ± 0.7% of the theoretical amount incorporated into the formulation (*n* = 3). Because the specificity of the method was not established and extraction recovery was not determined, this value is reported as an apparent content that may include a contribution from co-extracted formulation components. A value close to the theoretical amount is consistent with efficient incorporation of the active substance.

### 2.9. Short-Term Physical Stability

The stability of the neomycin sulfate-loaded emulgel was evaluated after two weeks under different temperature conditions. The freshly prepared formulation was used as the reference sample, while the other samples were stored at room temperature, under refrigerated conditions, or exposed to heating followed by storage at room temperature. The evaluation included macroscopic examination, viscosity, pH, spreadability, and neomycin sulfate content. The samples stored at room temperature and under refrigerated conditions maintained a homogeneous, white, semisolid appearance, with no visible phase separation or aggregation. By contrast, the heat-treated sample developed a slight yellowish color, although no clear phase separation was observed. This change may be related to temperature-induced alterations in the oily phase, surfactant system, or polymeric network. The heat-treated sample also showed the largest change in the value obtained by the UV method. Because this sample had become yellowish and the method's specificity was not established, this change is reported as an apparent decrease in neomycin sulfate content. It may reflect degradation of the active substance, but it may equally reflect an altered spectral background arising from colored products formed in the oily phase or in the surfactant system. The two contributions cannot be separated from the method used here; therefore, the result is presented as an indication that the formulation is thermally sensitive rather than as a quantified measure of drug degradation.

**Figure 3:**
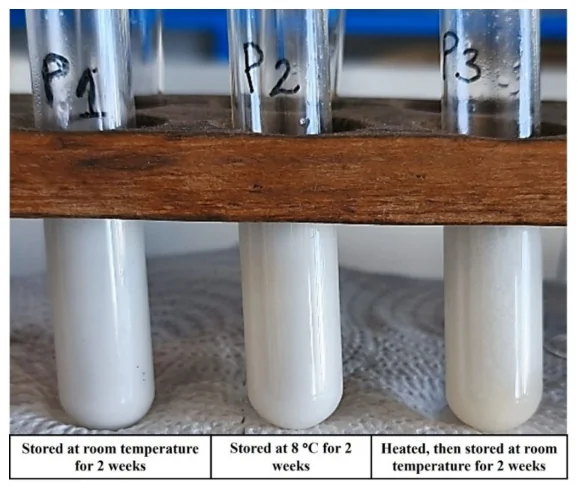
Macroscopic appearance of the neomycin sulfate-loaded emulgel after two weeks of storage: (P1) room temperature; (P2) refrigerated conditions, 8 °C; (P3) heat treatment followed by storage at room temperature. Refrigeration did not cause visible destabilization, although changes in viscosity, spreadability, and apparent drug content suggested that low temperature influenced the formulation's internal organization. The heat-treated sample exhibited the most pronounced modifications, including changes in rheological behavior, spreadability, appearance, and a marked reduction in the apparent neomycin sulfate content.

The physicochemical results showed only minor variations at room temperature compared with the initial formulation. Given the two-week duration and the absence of replicate storage batches, these results are reported as preliminary short-term observations rather than as evidence of formulation stability (Figure 3).

**Figure 4:**
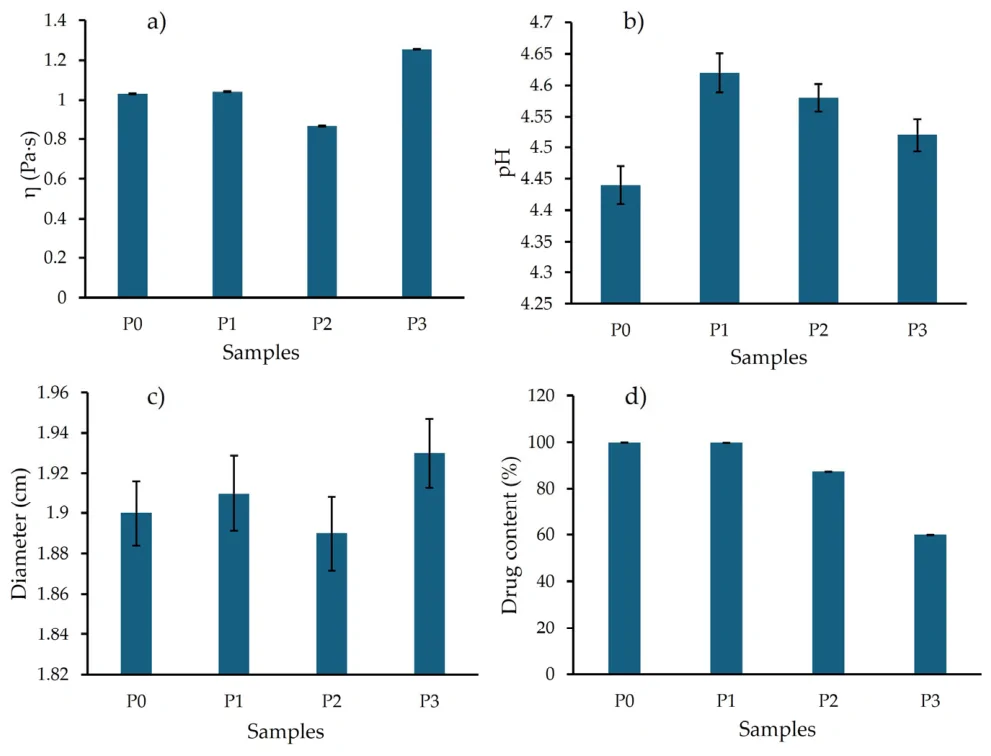
Comparative variation of (**a**) viscosity, (**b**) pH, (**c**) spreadability, and (**d**) neomycin sulfate content of the emulgel under different storage conditions. P0, freshly prepared reference sample; P1, stored at room temperature; P2, stored at approximately 8 °C; P3, heated and subsequently stored at room temperature.

The comparative variations in viscosity, pH, spreadability, and drug content are presented in Figure 4. Overall, room-temperature storage best preserved the physical characteristics and active substance content of the emulgel, whereas exposure to elevated temperatures had the greatest negative impact on formulation stability.

### 2.10. In Vitro Release Profile of Neomycin Sulfate

The in vitro release of neomycin sulfate from the emulgel was evaluated using a Franz-type diffusion cell. All measurements were obtained from a single Franz cell, sampled sequentially at the seven time points; no independent replicate cells were used. The donor compartment was not occluded during the experiment, so evaporative water loss from the emulgel over the 120 min test cannot be excluded; this would concentrate the formulation in the donor phase over time and could contribute to the observed release profile independently of the polymeric network.

Approximately 1 g of emulgel containing 0.1% neomycin sulfate was placed in the donor compartment, while phosphate-buffered solution at pH 7 was used as the receptor medium. The inner eggshell membrane was used as a semipermeable diffusion barrier. The system was maintained at 37 °C under continuous stirring at 100 rpm. Samples were collected from the receptor medium at predetermined time intervals: 5, 10, 20, 30, 60, 90, and 120 min. After each sampling, the removed volume was replaced with fresh receptor medium to maintain a constant volume. The concentration of neomycin sulfate was calculated using the calibration equation, and the cumulative amount released was corrected for the amount of drug removed during previous sampling. The in vitro release parameters of neomycin sulfate from the emulgel are presented in Table 4.

**Figure 5:**
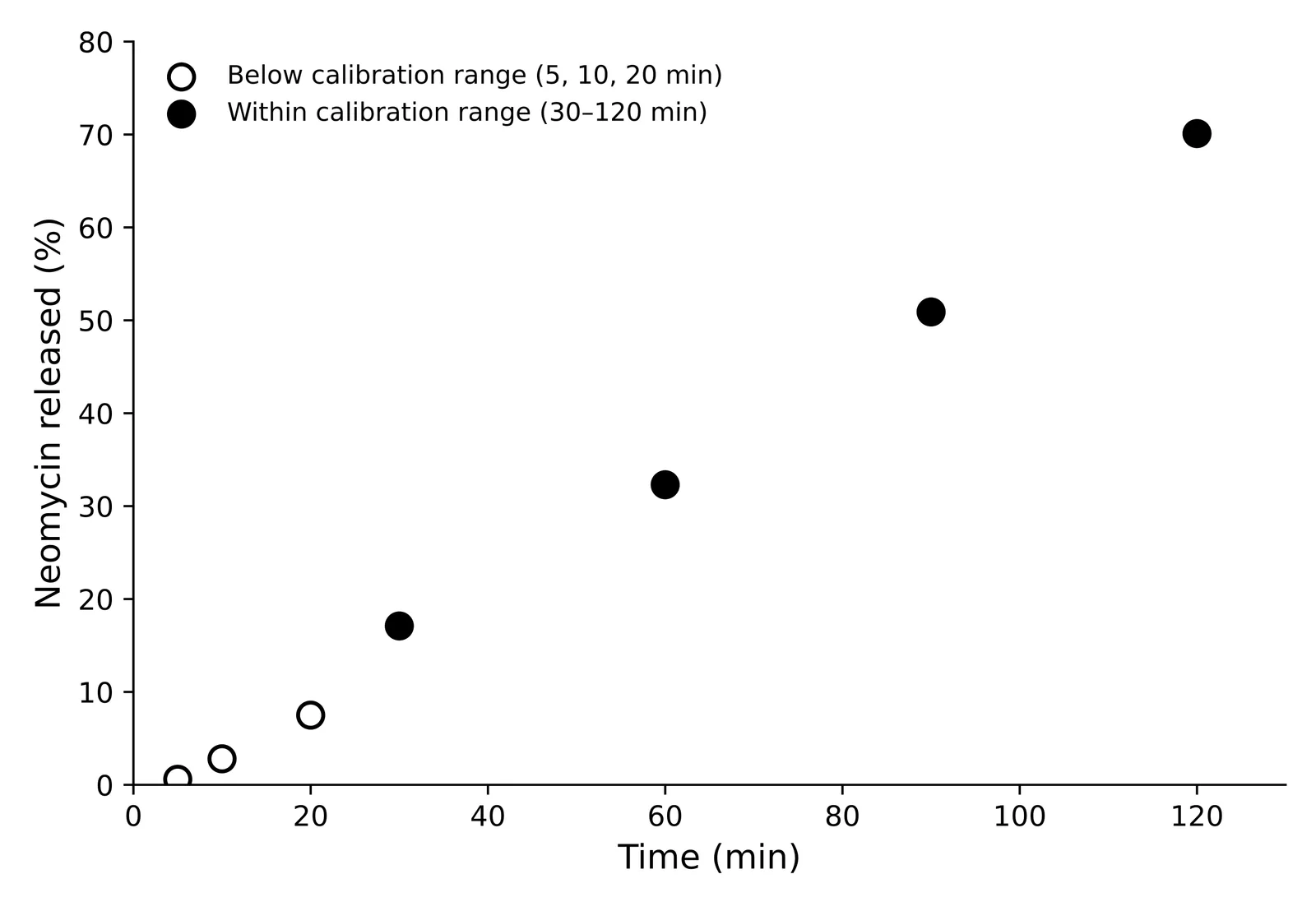
In vitro release profile of neomycin sulfate from the polymeric oil-in-water emulgel, showing the progressive increase in drug release over time.

The release profile showed a gradual increase in neomycin sulfate release over time (Figure 5). The concentrations measured at 5, 10, and 20 min were below the lowest calibration standard (5 µg/mL), and the 5 min value was additionally below the limit of quantification of the method (LOQ = 0.388 µg/mL); these three points are marked in Table 4 and are reported as indicative rather than quantitative. These three concentrations were obtained by extrapolation below the lowest calibration standard, and they are therefore reported as estimates rather than as quantitative determinations. They are retained in Table 4 for completeness of the profile, and their influence on the kinetic analysis is assessed separately in Section 2.11. From 30 min onward, all measured concentrations fell within the established calibration range. The cumulative release reached 17.1% after 30 min, 32.3% after 60 min, and 50.9% after 90 min, and approximately 70.1% of the theoretical drug content was released after 120 min. Even under the deviations observed for this method in the corresponding concentration region, up to 26% below 20 µg/mL, the resulting uncertainty in the cumulative amount released at 120 min does not exceed 15.4 µg, that is, 2.2% of the total (701.2 µg); the release profile from 30 min onward and the kinetic analysis are unaffected.

This behavior is consistent with a contribution of the chitosan–methylcellulose matrix contributing to neomycin sulfate diffusion. However, the release process in this experimental setup was governed jointly by the emulgel matrix and by the eggshell membrane used as the diffusion barrier in the Franz cell. Because the study did not include a membrane-free or matrix-free control, the relative contribution of each element to the observed release profile cannot be determined from the present data. Neomycin sulfate is highly hydrophilic and ionizable, which would normally favor rapid diffusion into the aqueous receptor medium; the slower, progressive profile observed here indicates that the combined matrix-membrane system imposed some resistance to drug transport, without allowing the two contributions to be distinguished.

Sink conditions were maintained throughout the experiment: the maximum measured concentration in the receptor medium was 27.79 µg/mL (corresponding to approximately 0.28% of the reported aqueous solubility of neomycin sulfate), which was below 1% of the reported aqueous solubility of neomycin sulfate in phosphate buffer (approximately 10 mg/mL at pH 7.2) [30]. The absence of a final plateau at 120 min indicates that release was still progressing when sampling ended. Given the short duration of the test and the unoccluded donor compartment, this supports a progressive release process but is not sufficient to establish prolonged or sustained topical delivery, which would require measurements over a longer interval under controlled donor conditions.

The release rate and flux were calculated using the following equations:

The release rate was calculated for successive time intervals using the following equation:

$$v=\frac{\Delta M}{\Delta t}$$

where:

v = release rate;

ΔM = change in the amount of neomycin released between two consecutive time points;

Δt = time interval.

The active substance release flux was also calculated using the following equation:

$$J=\frac{\Delta M}{S\cdot \Delta t}$$

where:

J = release flux;

S = diffusion area.

The diffusion area was calculated from the radius of the active membrane region using the equation $S=\pi r^{2}$ . Since the radius was 1.2 cm, the calculated diffusion area was 4.52 cm^2^. The results are presented in Table 5.

The release rate increased during the first 30 min, reaching the highest value in the 20–30 min interval. This may indicate progressive hydration of the polymeric matrix and enhanced mobilization of neomycin sulfate within the emulgel. After this interval, the release rate remained moderate, suggesting that the process was controlled by diffusion through the semisolid matrix and possibly by polymer relaxation phenomena. The flux values followed a similar trend, confirming that the release process was not instantaneous but modulated by the structure of the polymeric emulgel.

### 2.11. Release Kinetics Modeling

**Figure 6:**
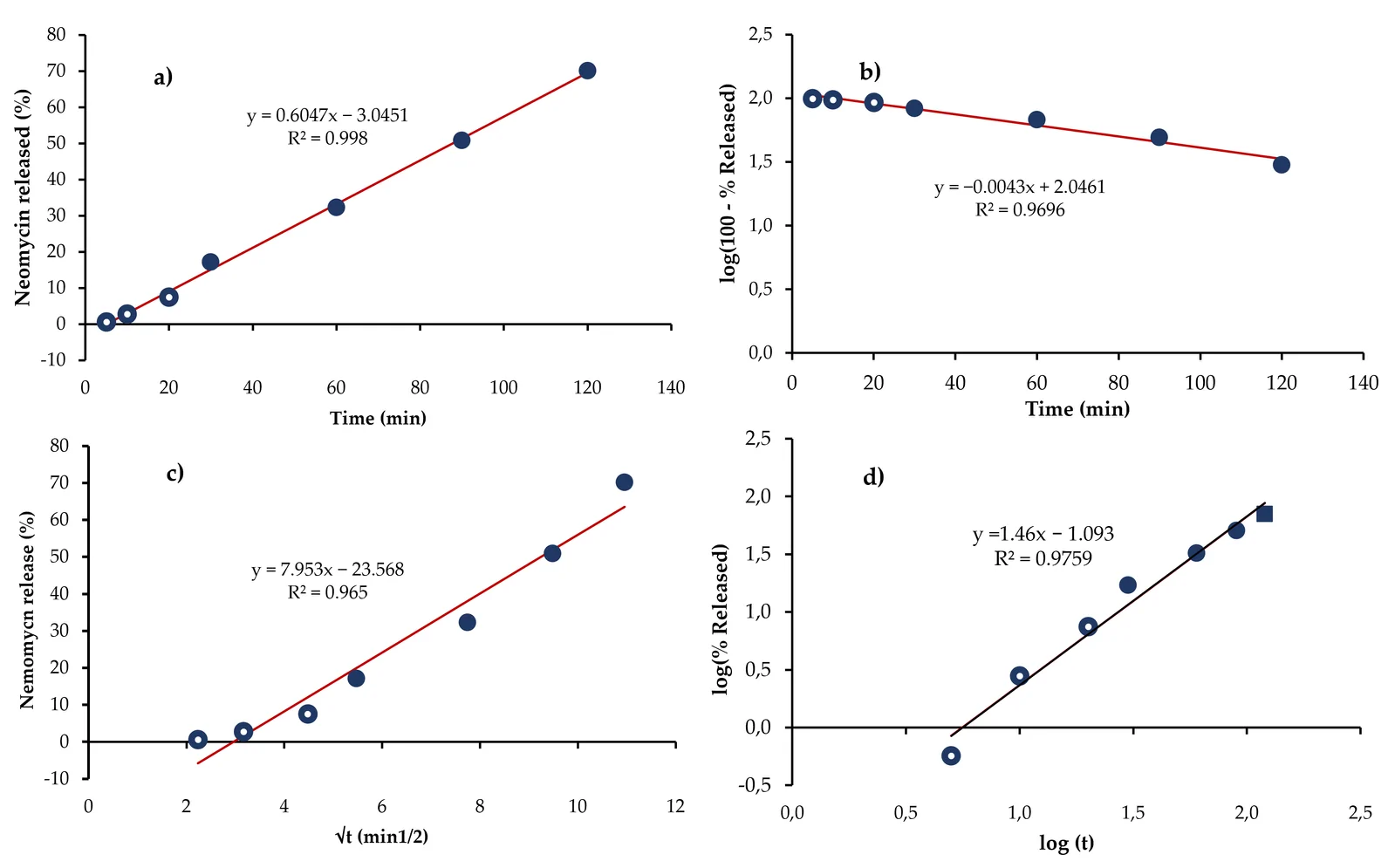
Kinetic modelling plots for the in vitro release of neomycin sulfate from the polymeric oil-in-water emulgel: (**a**) zero-order model; (**b**) first-order model; (**c**) Higuchi model; (**d**) Korsmeyer–Peppas model. Open circles denote the 5, 10, and 20 min points, below the established calibration range of the analytical method (Section 2.10); filled circles denote the 30 to 120 min points. In (**d**), all seven sampling points were included in the regression. The square denotes the 120 min point, which corresponds to 70.1% released and therefore lies above the customary applicability limit of the model.

To characterize the release process of neomycin sulfate from the polymeric emulgel, experimental data from the diffusion study were analyzed using zero-order, first-order, Higuchi, and Korsmeyer–Peppas kinetic models. The kinetic modeling plots describing neomycin sulfate release from the polymeric oil-in-water emulgel are presented in Figure 6. For kinetic modeling, the cumulative amount of neomycin released (M_cum_) was used. This value was determined by applying the correction for successive sample withdrawals and replacement of the receptor medium after each sampling, according to the following equation:

$$M_{cum}=C_{t}\cdot V+\sum C_{i}\cdot V_{s}$$

where $M_{cum}$ represents the cumulative amount released $,C_{t}$ is the concentration determined at the current sampling time, V is the total volume of the receptor medium, $C_{i}$ represents the concentrations of the previously collected samples, and $V_{s}$ is the volume of the withdrawn sample.

To characterize the release process of neomycin sulfate from the polymeric emulgel, the experimental data obtained from the diffusion study (Table 4) were fitted to zero-order, first-order, Higuchi, and Korsmeyer–Peppas kinetic models. All four models were fitted to the same seven sampling points, so that R^2^, RMSE, and AIC refer to a common data set. For each model, the appropriate linear transform was regressed against time or log time, and the metrics were computed after back-transforming the model predictions to the percentage-released scale, so that models using different linear transforms remain directly comparable. The Korsmeyer–Peppas model was additionally fitted to the 5 to 90 min interval, corresponding to less than approximately 60% of the drug released, in accordance with the customary applicability range of the model [31]. That restricted fit is reported as a sensitivity analysis and is not used for model ranking. Model parameters are summarized in Table 6, and the corresponding plots are shown in Figure 6.

The zero-order model provided the best description of the release process (R^2^ = 0.9980), followed by the first-order (R^2^ = 0.9697) and Higuchi (R^2^ = 0.9650) models, which performed similarly to each other; the Korsmeyer–Peppas model gave the weakest fit (R^2^ = 0.9077). The same ranking was obtained by RMSE and by AIC, both computed on the same seven observations (Table 6). This ranking describes the shape of the measured profile and is not taken as evidence of a constant-rate, matrix-controlled release process. The fitted zero-order line has an intercept of −3.0% and crosses zero at approximately 5 min, which has no physical counterpart in the release process and shows that a straight line does not describe the earliest part of the profile. The largest residuals occur at 20 and 30 min, −1.6 and +2.0 percentage points, close to the lower boundary of the validated analytical range; since the three earliest points lie outside that range (Section 2.10), the departure from linearity in this region cannot be separated from analytical uncertainty. Restricting the fit to the 30 to 120 min interval reduces the intercept to −1.8% and leaves the ranking unchanged (Table 7). The zero-order model is therefore reported as the best empirical description of the measured interval rather than as a demonstrated release mechanism. The release rate may nonetheless be influenced by formulation-related factors such as the polymeric phase, viscosity, and organization of the semisolid system, as reported for other topical emulgels [22], and the Higuchi and first-order models describe the data adequately without fitting decisively better than the constant-rate model.

The Korsmeyer–Peppas fits were computed as a sensitivity analysis rather than as alternative mechanistic descriptions of the release process: *n* = 1.46 on all seven sampling points, *n* = 1.52 on the 5 to 90 min interval, and *n* = 1.02 on the four points within the validated calibration range, 30 to 120 min. The exponent therefore varies by approximately 0.5 depending on which sampling points are included, and the first of these fits extends beyond the release fraction for which the model is usually considered applicable. None of the three values is used to assign a transport mechanism. The zero-order model remains the best fit on each of the three data sets, with R^2^ = 0.9980, 0.9961, and 0.9972, respectively.

To assess the influence of the three sampling points that fall outside the validated analytical range, all four models were refitted to the 30 to 120 min interval, in which every measured concentration lies within the calibration range (Table 7).

R^2^ and RMSE were computed on the percentage-released scale after back-transformation. AIC is not reported for the four-point fits, since with four observations and three estimated parameters the criterion is not sufficiently reliable for model discrimination.

The ranking of the models is unchanged: the zero-order model gives the best description of the release process on both data sets, and the reduction in R^2^ from 0.9980 to 0.9972 is marginal. The release exponent of the Korsmeyer–Peppas model, by contrast, is strongly affected, falling from 1.46 to 1.02. As set out above, this variation is reported as a sensitivity analysis and no transport mechanism is assigned to either value.

The near-linear cumulative profile observed between 5 and 120 min is compatible with several of the release processes described for emulgel and hydrogel-based systems [24,32], and the present design does not allow them to be distinguished. The zero-order model is used here as an empirical description of the measured interval, not as evidence that release is controlled by the chitosan–methylcellulose matrix.

## 3. Conclusions

A polymeric oil-in-water emulgel containing neomycin sulfate was successfully formulated using chitosan and methylcellulose as gelling agents. The formulation showed suitable characteristics for topical application, including a homogeneous semisolid appearance, good spreadability, mildly acidic pH, and efficient incorporation of the active substance. Microscopic analysis confirmed the oil-in-water structure of the emulgel and showed that ultrasonication improved the uniformity of the dispersed phase. The rheological behavior over the tested range, 50 to 100 rpm, was pseudoplastic, with a flow behavior index of 0.19, although behavior at true rest was not measured. The in vitro release study showed a progressive release of neomycin sulfate over 120 min, with the zero-order model providing the best fit among the models tested over that interval. Since a single formulation was evaluated without a placebo emulgel, a free-drug solution, or a comparator, this profile characterizes the combined emulgel and membrane system and cannot be attributed to the chitosan–methylcellulose network as such. The study is therefore presented as a proof-of-concept investigation, and the results provide a preliminary basis for further formulation work rather than evidence of a release-modulating effect. The quantitative data reported here were obtained by direct UV spectrophotometry, a method that was not validated for specificity in the present formulation matrix; the corresponding values should therefore be regarded as apparent and as suitable for internal comparison rather than as validated assay results. Future studies should include placebo and comparator formulations, formulation optimization, extended stability testing, ex vivo permeation assays, antimicrobial evaluation, and skin compatibility studies.

## 4. Materials and Methods

### 4.1. Materials

Neomycin sulfate, pharmaceutical-grade methylcellulose, chitosan with a medium molecular weight and a degree of deacetylation of approximately 75–85%, and Tween 80 were purchased from Sigma-Aldrich/Merck, Darmstadt, Germany. Acetic acid of analytical grade was obtained from Chimopar, Bucharest, Romania. Sweet almond oil was purchased from Adams Vision SRL, Târgu Mureș, România. Distilled water was used throughout all experiments.

### 4.2. Equipment

The preparation and homogenization of the emulgel were performed using a thermostated magnetic stirrer from Velp Scientifica, Usmate Velate, Italy. Ultrasonication was carried out using a Bandelin ultrasonic homogenizerBerlin, Germany, to improve the dispersion and homogeneity of the formulation. The components were weighed using a Sartorius LE 623 P analytical balance, Göttingen, Germany. The pH and electrical conductivity measurements were performed using a Consort C862 multiparameter device, Turnhout, Belgium. Rheological analysis was carried out using an IKA ROTAVISC rotational viscometer, Staufen im Breisgau, Germany, equipped with spindle 9. UV–Vis spectrophotometric analyses were performed using an Infitek SP-LUV 7600 UV–Vis spectrophotometer Shanghai, China, equipped with quartz cuvettes. The in vitro release study was conducted using a Franz diffusion cell.

### 4.3. Preparation of the Neomycin Sulfate Emulgel

The emulgel was formulated as an oil-in-water system containing neomycin sulfate as the active pharmaceutical ingredient, chitosan and methylcellulose as polymeric gelling agents, Tween 80 as an emulsifier, and sweet almond oil as the oily phase.

**Figure 7:**
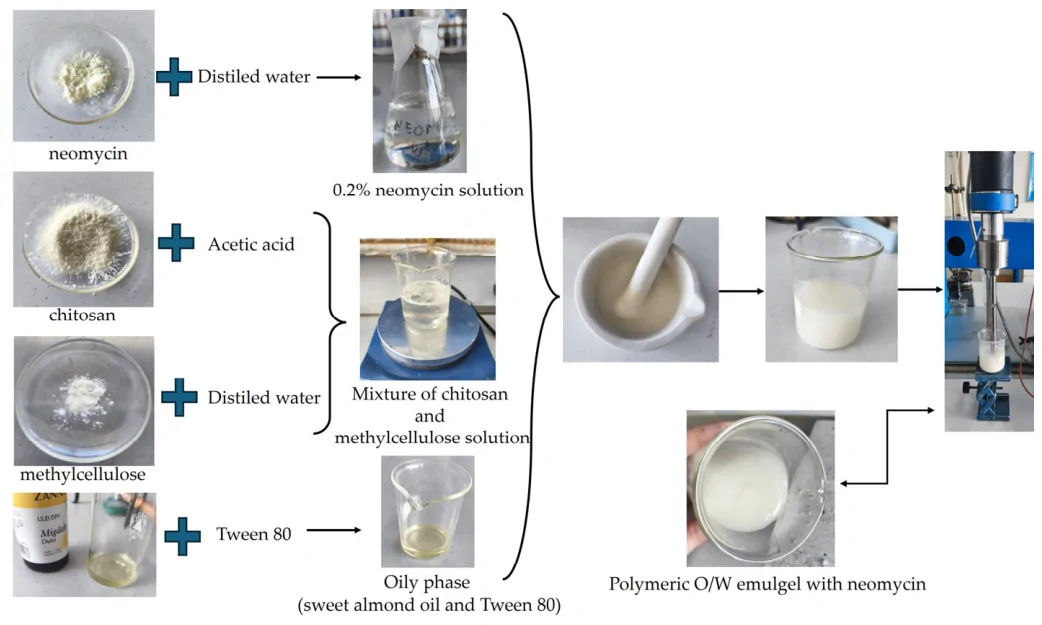
Preparation steps of the neomycin-loaded emulgel.

The composition of the formulation was as follows: neomycin sulfate 0.1 g, chitosan 1.0 g, acetic acid solution 0.5 M 5.0 g, methylcellulose 1.5 g, Tween 80 3.0 g, sweet almond oil 7.0 g, and distilled water up to 100.0 g. Chitosan was dispersed in 5.0 g of 0.5 M acetic acid solution under continuous stirring at 500 rpm for 3 h, until a homogeneous polymeric dispersion was obtained. Separately, methylcellulose was gradually dispersed in approximately 20 mL of cold distilled water under vigorous stirring to avoid aggregate formation and was then left to hydrate for 30 min. Neomycin sulfate was dissolved in approximately 50 mL of distilled water under stirring until a clear solution was obtained. The chitosan dispersion was incorporated into the hydrated methylcellulose gel under continuous stirring, followed by the addition of the neomycin sulfate solution, resulting in a homogeneous aqueous polymeric phase. The oily phase was prepared separately by mixing sweet almond oil with Tween 80 until complete homogenization. This phase was gradually added to the aqueous phase under moderate stirring. The formulation was then adjusted to a final mass of 100 g with distilled water and homogenized at 900 rpm for 1 h. Finally, the emulgel was ultrasonicated for 3 min and left to stand for 24 h to remove air bubbles and allow system stabilization. The preparation steps of the neomycin-loaded emulgel are schematically presented in Figure 7.

### 4.4. Organoleptic Evaluation

The prepared emulgel was evaluated macroscopically in terms of appearance, color, odor, consistency, homogeneity, and spreadability. The presence of phase separation, visible aggregates, or non-uniform areas was also recorded. The evaluation was performed on three independently prepared samples.

### 4.5. pH Determination and Electrical Conductivity Measurement

The pH of the emulgel was determined after dispersing 1 g of formulation in 10 mL of distilled water. The dispersion was homogenized and left to equilibrate at room temperature before measurement. The pH was determined by dispersing the emulgel in distilled water, following procedures reported for the characterization of semisolid gel and emulgel formulations. The conductivity of the emulgel was determined to evaluate the mobility of ionic species present in the formulation. For the analysis, an aqueous dispersion was prepared by mixing 1 g of emulgel with 10 mL of distilled water, a method also reported in the literature [24,33].

### 4.6. Optical Microscopy

The internal structure and homogeneity of the emulgel were evaluated by optical microscopy before and after ultrasonication. Microscopic observations were used to assess the distribution of the dispersed oily phase and the effect of ultrasonication on droplet size and uniformity. Methylene blue was used as a hydrophilic dye to identify the continuous aqueous phase and to confirm the oil-in-water character of the emulgel. Microscopic evaluation was performed in triplicate using three independently prepared samples.

### 4.7. Determination of Hydration Capacity

The hydration capacity of the neomycin sulfate-loaded polymeric oil-in-water emulgel was evaluated gravimetrically in phosphate-buffered solution. Considering the oil-in-water structure of the formulation, with an aqueous continuous phase structured by chitosan and methylcellulose, this test was interpreted as water uptake behavior rather than swelling capacity in the classical sense. A known amount of emulgel was accurately weighed (W0) and placed in contact with phosphate-buffered solution at 37 °C. At predetermined time intervals, the sample was removed, the excess surface liquid was gently blotted with filter paper, and the hydrated sample was weighed again (Wt). The hydration index was calculated according to the following equation:

$$HI\left(\%\right)=\frac{W_{t}-W_{0}}{W_{0}}\times 100$$

where HI represents the hydration index, W0 is the initial weight of the emulgel, and Wt is the weight of the emulgel after contact with the receptor medium at time t.

### 4.8. Rheological Analysis

The rheological behavior of the emulgel was evaluated using an IKA ROTAVISC rotational viscometer equipped with spindle 9.

Measurements were performed at room temperature (20 °C) at rotational speeds ranging from 50 to 100 rpm, in increments of 10 rpm. The apparent viscosity was recorded in Pa·s. Shear rate and shear stress were not calculated: according to the manufacturer, this instrument outputs shear rate and shear stress only with a coaxial, concentric-cylinder measuring system, and no conversion factor is published for the standard spindle set, nor was one established by independent calibration in this work. The flow behavior of the emulgel was assessed by fitting apparent viscosity as a function of rotational speed to the power-law equation η = K′·N^n−1^, where n is the flow behavior index, mathematically equivalent to the exponent of the Ostwald–de Waele model (τ = Kγ^ṅ^) independently of the unknown shear-rate proportionality constant.

### 4.9. Spreadability

Spreadability is an important quality attribute of topical formulations, as it reflects their ability to be applied uniformly to the skin and is closely related to the product's rheological properties. Adequate spreadability facilitates easy application and ensures even distribution of the formulation in the treated area. For its determination, 0.1 g of the sample was placed between two glass plates measuring approximately 5 × 5 cm, and a 50 g load was applied for 1 min, following a previously reported method [34]. The sample diameter was measured before load application and again after load removal. The corresponding spreading areas were calculated using the equation $S=\pi d^{2}/4$ , where S is the spreading area and d is the measured diameter. The initial diameter was determined using three separately deposited samples, whereas the diameter after load application was measured once.

### 4.10. Physical Stability Study

The physical stability of the emulgel was evaluated after 2 weeks of storage under different temperature conditions. Approximately 5 mL of the freshly prepared emulgel was retained as the reference sample (P0). The remaining emulgel was transferred into test tubes and divided into three groups: P1, stored at room temperature (20 °C); P2, stored at approximately 8 °C; and P3, heated in a water bath at 70 °C for 2.5 min and subsequently stored at room temperature (20 °C). All tubes were kept under ambient laboratory light, protected from direct sunlight. Each condition, P0 to P3, consisted of a single tube, from which repeated readings were taken; independent storage replicates were not prepared. After the storage period, the samples were visually examined for changes in color, consistency, homogeneity, aggregation, and phase separation.

### 4.11. UV–Vis Calibration Curve for Neomycin Sulfate

Neomycin sulfate lacks a strong conventional chromophore because its structure does not contain aromatic or extended conjugated systems. Nevertheless, aqueous neomycin sulfate solutions exhibit a weak absorption band in the near-UV region. An absorption maximum at 304.8 nm has previously been reported and associated with concentration-dependent molecular self-association and monomer–dimer equilibria [35]. In the present study, the spectra of all standard solutions were recorded between 200 and 400 nm. A reproducible local maximum was observed at 300±3 nm (mean ± SD, n=8 ), preceded by a local minimum at approximately 256 nm, and the absorbance at this wavelength increased with concentration (Figure 2a). Because the electronic origin of this band has not been definitively established and the cited study used substantially higher concentrations, 300 nm was selected as an experimentally identified absorption maximum under the conditions of the present work.

For calibration, a 1 mg/mL neomycin sulfate stock solution was prepared in phosphate buffer,p(H 7,)and serially diluted to obtain standards iranging from5–100 µg/mL. A phosphate-buffered solution pH 7 was used as a blank. The calibration curve was obtained by plotting absorbance aversusneomycin sulfate concentration. The resulting linear regression equation was used to calculate the concentration of neomycin sulfate in the release samples. The limit of detection (LOD) and limit of quantification (LOQ) were calculated using the equations LOD = 3.3σ/S and LOQ = 10σ/S, where S is the slope of the calibration curve, and σ is the standard deviation of five replicate absorbance readings recorded at 300 nm at the low end of the analytical response (mean 0.0151, SD 1.10 × 10^−4^ absorbance units). All spectrophotometric measurements for the calibration standards, the drug-content sample, and the release samples were performed in triplicate.

### 4.12. Determination of Neomycin Sulfate Content

To determine the active substance content, 1 g of the neomycin sulfate emulgel was dispersed and diluted to 100 mL with PBS, pH 7. The sample was thoroughly homogenized and subsequently filtered through filter paper. The absorbance of the filtrate was measured spectrophotometrically at 300 nm, and the neomycin concentration was calculated using the calibration curve equation. The active substance content was expressed as a percentage by relating the experimentally determined amount of neomycin to the theoretical amount present in the formulation:

$$\mathrm{Neomycin}\;\mathrm{content}\;(\%)\;=\frac{\mathrm{experimentally}\;\mathrm{determined}\;\mathrm{amount}}{\mathrm{theoretical}\;\mathrm{amount}}\times 100$$

### 4.13. In Vitro Release Study

The in vitro release of neomycin sulfate from the emulgel was investigated using a Franz-type diffusion cell. Approximately 1 g of emulgel was placed in the donor compartment and uniformly distributed on the surface of the diffusion membrane. The inner membrane of the eggshell was excised and cut to the size of the diffusion cell aperture, then hydrated in the receptor medium before use. Membrane thickness and integrity were not assessed. The donor compartment was left uncovered, open to ambient air, for the duration of the experiment. The selection of this membrane type was justified by previous literature reports describing its use in vitro diffusion studies for assessing the release of active substances from gels, patches, and other topical delivery systems [36,37].

The receptor compartment was filled with 20 mL of PBS, pH 7. The system was maintained at 37 °C and continuously stirred at 100 rpm to ensure uniform distribution of the released drug in the receptor medium. Samples of approximately 3 mL were collected from the receptor medium at predetermined time intervals: 5, 10, 20, 30, 60, 90, and 120 min. After each sampling, the same volume of fresh receptor medium, maintained at 37 °C, was added to keep the receptor volume constant. The collected samples were analyzed by UV–Vis spectrophotometry at 300 nm. The concentration of neomycin sulfate released at each time point was calculated using the calibration curve. The cumulative amount released was corrected by considering the amount of drug removed during previous samplings.

### 4.14. Release Kinetics Modeling

The experimental release data were fitted to mathematical models commonly used to describe drug release from semisolid and polymeric systems: zero-order, first-order, Higuchi, and Korsmeyer–Peppas models.

The zero-order model was expressed as:

$$M_{t}=M_{0}+k_{0}t$$

where M_t_ is the cumulative amount of drug released at time t, M_0_ is the initial amount of drug released, and k_0_ is the zero-order release constant.

The zero-order kinetic model describes a situation in which the active substance is released at a constant rate, independently of the amount remaining in the formulation. This type of release is characteristic of systems designed to maintain a constant delivery of the active substance over time [38].

The first-order model was expressed as:

$$log(100-M_{t})=logM_{0}-\frac{k_{1}t}{2.303}$$

where $k_{1}$ is the first-order release constant.

The first-order kinetic model assumes that the release rate depends on the amount of active substance remaining in the formulation [39]. In this case, the rate of the process progressively decreases as the amount of neomycin available in the matrix diminishes.

The Higuchi model was expressed as:

$$M_{t}=k_{H}t^{1/2}$$

where $k_{H}$ is the Higuchi release constant.

The Higuchi model is frequently used to describe the release of active substances from semisolid systems and polymeric matrices, where transport is predominantly controlled by diffusion [40].

The Korsmeyer–Peppas model is used to investigate complex release mechanisms that may simultaneously involve diffusion, hydration, and relaxation of polymer chains, and its applicability has often been reported for emulgel formulations [24,41].

The Korsmeyer–Peppas model was expressed as:

$$\frac{M_{t}}{M_{\infty }}=kt^{n}$$

and, after logarithmic transformation:

$$log\left(\frac{M_{t}}{M_{\infty }}\right)=logk+nlogt$$

where $M_{t}/M_{\infty }$ is the fraction of drug released at time t , k is the kinetic constant; and *n* is the release exponent. For this model, only the initial part of the release profile, corresponding to less than approximately 60% drug release, was considered.

### 4.15. Statistical Analysis

Except for the organoleptic and microscopic analyses, the experimental determinations were performed in triplicate using repeated readings or samples collected from the same prepared formulation batch. The results were expressed as mean ± standard deviation. Data tabulation and descriptive statistics were performed using Microsoft Excel for Microsoft 365 (Microsoft Corporation, Redmond, WA, USA). Regression fitting of the kinetic models, back-transformation to the percentage-released scale, and calculation of R^2^, RMSE, AIC, and propagated uncertainties were performed in Python 3.11 using NumPy 2.4.4 and SciPy 1.17.1.

## Figures and Tables

**Table 1:** Organoleptic characteristics of the neomycin sulfate-loaded emulgel.

Evaluated Parameter	Observation
Appearance	Semisolid, creamy, homogeneous
Color	White
Odor	Weak characteristic odor
Consistency	Soft, gelatinous, viscous, easy to sample
Homogeneity	No visible phase separation or large aggregates
Spreadability	Good; uniform layer formation
Topical acceptability	Suitable from an organoleptic point of view

**Table 2:** Hydration index of the neomycin sulfate-loaded oil-in-water emulgel in phosphate-buffered solution.

Time (min)	Initial Weight, (W_0_) (g)	Hydrated Weight, (W_t_) (g)	Hydration Index, (HI) (%)
5	1	1.025	2.5
10		1.041	4.1
20		1.066	6.6
30		1.089	8.9
60		1.132	13.2
90		1.164	16.4
120		1.185	18.5

**Table 3:** Rheological parameters of the neomycin sulfate-loaded emulgel, measured at 20 °C.

Rotational Speed (rpm)	Spindle	*η* (Pa·s) ± SD
50	9	1.032 ± 0.003
60		0.931 ± 0.0025
70		0.845 ± 0.015
80		0.775 ± 0.0021
90		0.680 ± 0.0015
100		0.572 ± 0.0026

Shear rate and shear stress are not reported, since no manufacturer-validated or independently calibrated conversion factor exists for this spindle (Section 4.8). Fitting apparent viscosity against rotational speed, *η = K′·N^n−1^*, gave a flow behavior index *n* = 0.191 (R^2^ = 0.957), confirming pronounced shear-thinning behavior over the tested range; this exponent is independent of the unvalidated shear-rate conversion. Only 50 to 100 rpm was tested, and measurements at lower speeds or under quiescent conditions were not performed, so the higher viscosity at the lowest tested speed is consistent with, but does not demonstrate, favorable behavior at rest. Overall, the formulation showed higher viscosity at low speed and a marked decrease at higher speed, consistent with, though not a direct measurement of, suitable behavior at rest and improved spreadability under applied stress.

**Table 4:** In vitro release parameters of neomycin sulfate from the emulgel.

Time (min)	C_t_ (µg/mL)	M_t_ = C_t_ × 20 (µg)	M_cum_ (µg)	Released Neomycin (%)
5 ^a^	0.29	5.7	5.7	0.6
10 ^b^	1.36	27.1	28.0	2.8
20 ^b^	3.50	70.0	74.9	7.5
30	7.79	155.7	171.1	17.1
60	14.21	284.3	323.1	32.3
90	21.36	427.1	508.6	50.9
120	27.79	555.7	701.2	70.1

^a^ Below the limit of quantification of the method (LOQ = 0.388 µg/mL); reported as indicative only. ^b^ Above the LOQ but below the lowest calibration standard (5 µg/mL), outside the established calibration range of the method (Section 2.7); reported as indicative only.

**Table 5:** Release rate and flux of neomycin sulfate from the emulgel.

Time (min)	M_cum_ (µg)	Δt (min)	ΔM_cum_ (µg)	v (µg/min)	J (µg/cm^2^·min)
5	5.7	5	5.7	1.14	0.25
10	28.0	5	22.3	4.46	0.99
20	74.9	10	46.9	4.69	1.04
30	171.1	10	96.2	9.62	2.13
60	323.1	30	151.9	5.06	1.12
90	508.6	30	185.5	6.18	1.37
120	701.2	30	192.6	6.42	1.42

**Table 6:** Kinetic model parameters for the in vitro release of neomycin sulfate from the emulgel.

Model	Equation	R^2^	RMSE	AIC
Zero order	% = 0.6047t − 3.043	0.9980	1.095	7.26
First order	log(100 − %) = −0.004346t + 2.0461	0.9697	4.269	26.32
Higuchi	% = 7.953√t − 23.565	0.9650	4.590	27.33
Korsmeyer–Peppas	1.460·log(t) − 1.093 (*n* = 1.46; k = 0.0807)	0.9077	7.452	34.12

All four models were fitted to the same seven sampling points (5 to 120 min). R^2^, RMSE, and AIC were computed on the percentage-released scale after back-transformation, so that models using different linear transforms remain comparable; on the log–log scale of Figure 6d, the same Korsmeyer–Peppas regression gives R^2^(log–log) = 0.9759. AIC was calculated as *n*·ln(RSS/*n*) + 2*p*, with *n* = 7 and *p* = 3 for all four models. Restricting the Korsmeyer–Peppas fit to the 5 to 90 min interval, six points corresponding to less than approximately 60% released, gives *n* = 1.52, k = 0.0689, and R^2^ = 0.8779; this restricted fit is reported as a sensitivity analysis and is not used for model ranking.

**Table 7:** Sensitivity of the kinetic analysis to the sampling points outside the validated analytical range.

Model	Full Data Set, 5 to 120 min (*n* = 7) R^2^	RMSE	Validated Range, 30 to 120 min (*n* = 4) R^2^	RMSE
Zero order	0.9980	1.095	0.9972	1.060
First order	0.9697	4.269	0.9520	4.357
Higuchi	0.9650	4.590	0.9770	3.015
Korsmeyer–Peppas ^a^	0.9077	7.452	0.9965	1.177

^a^ Release exponent *n* = 1.46 on the full data set and *n* = 1.02 on the established calibration range. On the validated range, the last sampling point corresponds to 70.1% released, above the customary 60% applicability limit of the model, which is a further reason for not using this exponent mechanistically.

## Data Availability

The data supporting the results reported in this study are available from the corresponding author upon reasonable request.
